# Product inhibition of secreted phospholipase A2 may explain lysophosphatidylcholines' unexpected therapeutic properties

**DOI:** 10.1186/1476-9255-5-17

**Published:** 2008-10-22

**Authors:** Timothy J Cunningham, Lihua Yao, Angel Lucena

**Affiliations:** 1 Neurobiology and Anatomy, Drexel University College of Medicine, Philadelphia, PA 19129, USA

## Abstract

**Background:**

Lysophosphatidylcholines (lysoPCs) are products of phospholipase A2 (PLA2) enzyme activity, and like the enzyme, have a direct role in toxic inflammatory responses in variety of organ systems. Paradoxically, reduced plasma lysoPC levels have been noted in sepsis patients and systemic treatment with lysoPCs is therapeutic in rodent models of sepsis and ischemia. These observations suggest that elevation of plasma levels of these lipids can actually help to relieve serious inflammatory conditions. We demonstrate that specific lysoPCs act as uncompetitive product inhibitors of plasma secreted PLA2 enzymes (sPLA2s), especially under conditions of elevated enzyme activity, thus providing a feedback mechanism for the observed anti-inflammatory effects of these compounds.

**Methods:**

Thin layer chromatography and mass spectroscopy were used to estimate total lysoPC concentration and the relative contributions of different lysoPC species in rat plasma samples. Kinetic studies of sPLA2 enzyme activity were conducted on these samples *ex vivo *and on purified group IA sPLA2 *in vitro *after addition of specific lysoPC species to the reaction mixture. Enzyme activity was also measured in plasma samples of rats injected with these same lysoPCs.

**Results:**

Palmitoyl (16:0), stearoyl (18:0) are the most abundant lysoPCs in rat plasma consistent with other reports. Kinetic studies demonstrated that both were uncompetitive inhibitors of plasma sPLA2 enzyme activity. *In vitro *experiments with group IA sPLA2 confirmed the inhibition and the kinetic properties of these lysoPC species. Decanoyl lysoPC (10:0), which was not detected in plasma, did not inhibit enzyme activity in vitro. LysoPC injections into normal rats resulted in "buffering" of plasma sPLA2 activity in a narrow low range, consistent with the activity-dependent inhibition suggested by the *ex vivo *and *in vitro *experiments.

**Conclusion:**

The results may explain the efficacy of lysoPC therapy during periods of elevated inflammatory activity and further highlight the utility uncompetitive enzyme inhibitors. In this case, the inhibitor is a product of the enzyme reaction, and therefore represents an example of activity-driven feedback inhibition.

## Background

Upregulation of circulating phospholipase A2 enzymes, principally the secreted isoforms (sPLA2s), is associated with the activity of the innate immune system and a number of inflammatory disorders [1-4]. Experimental and correlative studies suggest increased levels of sPLA2s in the blood contribute to or are predictive of the tissue destruction that occurs following trauma, heart and lung disease, local and systemic infections, brain damage, and autoimmune disorders [4-9]. PLA2 enzyme activity and the lipid mediators regulated by that activity are directly linked to a variety of cell death effector mechanisms including the production of reactive oxygen species (both directly and via inflammatory cells), excitotoxicity, and expression of the death receptor family members in a variety of cells [10-12]. Many of these activities have been studied relative to the fatty acid branch of PLA2 pathways, which includes arachidonic acid and its metabolites (prostanoids, leukotrienes)[13]. The other products of PLA2 hydrolysis, the lysophosphatides, are also potent biological mediators but their mechanisms are more enigmatic and their actions are often contradictory[14]. There is current interest in lysophosphatidylcholines (lysoPCs) in particular, as some of these are proposed for treatment of systemic inflammatory disorders. This suggestion is based on reports that plasma lysoPC levels are diminished with the onset of sepsis in human patients [15,16], and in rodent models of sepsis and ischemia, lysoPC treatment is an effective therapy [17-21].

In previous studies, we investigated sPLA2 enzyme activity in plasma of rats and humans because inhibition of this group of enzymes, and subsequent tissue repair and protection, is a long-standing goal of pharmacotherapeutics [22]. Our studies were expedited by the fact that plasma sPLA2 enzymes will follow Michaelis-Menten kinetics when incubated with a broad-spectrum substrate, even though the specific classes of sPLA2s active in plasma are diverse. This property made it possible to characterize a peptide inhibitor of sPLA2 activity (called CHEC-9), also with broad-spectrum activity, as well as demonstrate the therapeutic potential of sPLA2 inhibition *in vivo*. CHEC-9 treatment of both traumatic and autoimmune models of neurodegeneration resulted in significant cytoprotection and reduction of cell-mediated inflammation [5,6,23]. These results have been supported by other experimental studies both in and outside of the nervous system. For example, infusion of sPLA2 substrate-like compounds reduces circulating sPLA2 activity and also has both neuroprotective and anti-inflammatory effects [24]. Conversely, transgenic models that express high systemic levels of group II or group V sPLA2s demonstrate pro-atherogenic pathologies and exaggerated lung disease[8,25,26].

Given the therapeutic advantages of sPLA2 inhibition, we considered the possibility that under certain conditions the lysoPC product of sPLA2 activity actually inhibits the enzyme, thus explaining one aspect of the paradoxical behaviour of these lipids. Product inhibition is a widely recognized phenomenon in enzymology [27], but one that has received scattered experimental attention with respect to regulation of enzyme activity in mammalian systems *in vivo *[28,29]. As for sPLA2, product inhibition of phosphatidylcholine hydrolysis was suggested to occur *in vitro *during experiments with venomous group IA sPLA2 [30,31]. However there were different conclusions in these studies as to the source of this inhibition. Furthermore, serum albumin binding to lysoPC was suggested to neutralize its inhibitory activity, a property that might limit the applicability of the results to systemic therapies given the sheer abundance of albumin in plasma. In the present paper, we first documented the principal lysoPC species in rat plasma relative to each other and to other abundant plasma lipids using thin layer chromatography and mass spectroscopy. Then the effects of exogenous lysoPCs were studied with plasma sPLA2 activity *ex vivo *and with group IA sPLA2 *in vitro*. Finally, lysoPCs were injected into rats to determine the influence of elevating systemic levels of these compounds on circulating sPLA2 activity. The results suggest that the two most abundant lysoPC species found in plasma are activity-dependent uncompetitive inhibitors of sPLA2 enzyme activity, even in the presence of albumin.

## Methods

### Sources of enzymes

Blood was obtained from the trunk of 32 female Sprague Dawley rats (200–250 g) after decapitation. Blood samples were treated with citrate-phosphate-dextrose anticoagulant (1:10, Sigma), and plasma prepared by centrifugation after which samples were frozen at -80° until used. For 16 of these animals, plasma samples were pooled from 4–6 rats for *ex vivo *enzyme assays. The other 16 rats received lysoPC treatments (see below). The Institutional Animal Care and Use Committee of Drexel University College of Medicine approved all specific procedures of this study. Purified group IA secreted phospholipase A2 from the venom of the Mozambique spitting cobra (Naja mossambica) was obtained from Sigma (St. Louis, MO).

### Lipid analysis

#### Thin layer chromatography

**Figure 1:**
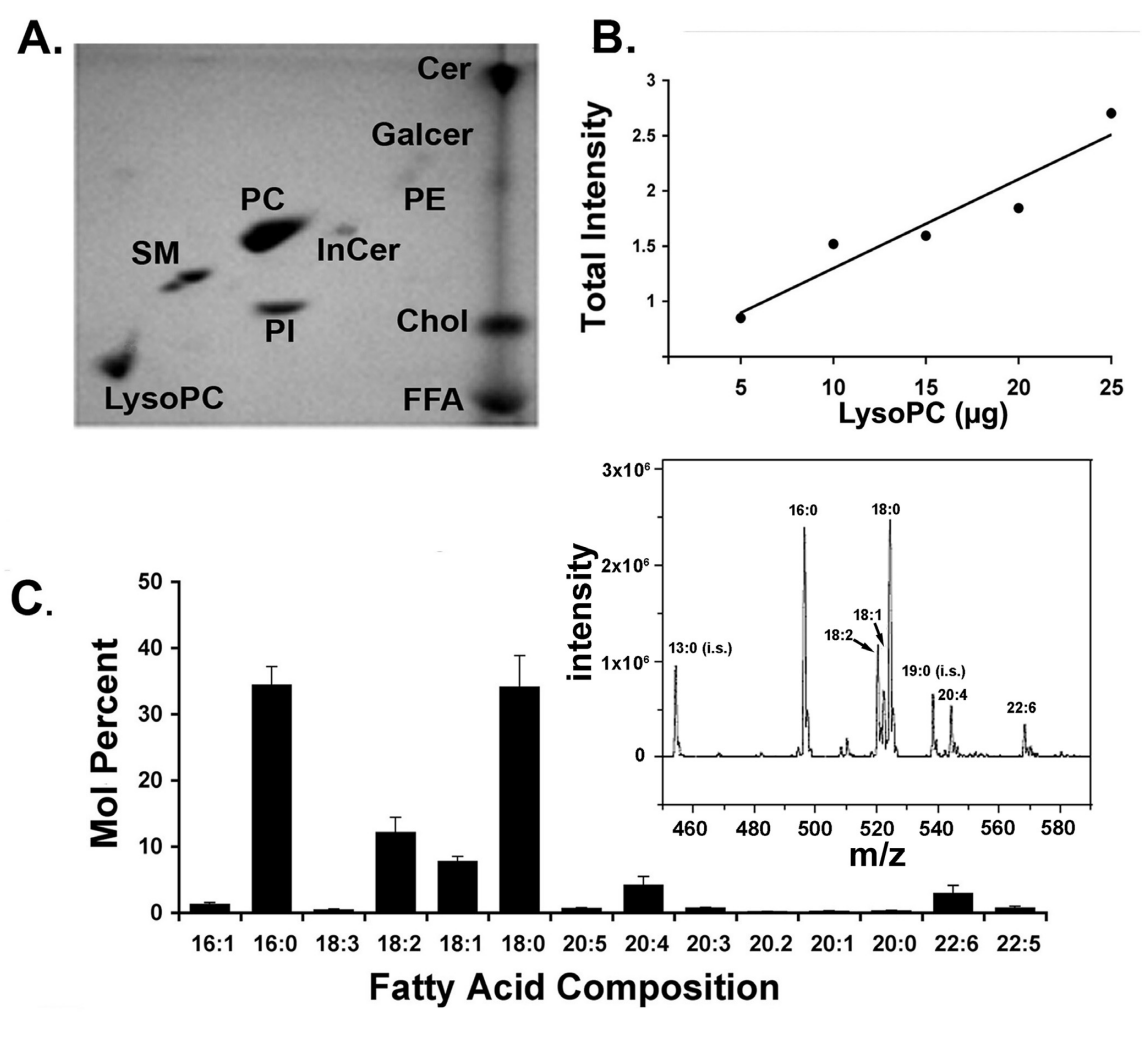
**Lysophosphatidylcholines in rat plasma**. (A.) Thin layer chromatography showing the lysoPC spot relative to other abundant plasma lipids. Abbreviations: Cer-ceramide; Galcer- galactoceramide; PC- phosphatidylcholine; PE- phosphatidylethanolamine; SM- sphingomyelin; PI- phosphatidylinositol. Chol- free cholesterol; FFA- free fatty acids; LysoPC- lysophosphatidylcholine. InCer- internal control. (B.) Standard curve prepared with a mixture of lysoPCs 16:0, 18:0, and 18:1 showing semi-quantitative nature of TLC method (see text). Total intensity of the lysoPC spots at different concentrations were normalized to the intensity of the internal control spot (Incer) developed on the same plate. (C.) Mol percent of individual species of lysoPCs in the samples calculated from mass spectra of plasma lipid extracts relative to total lysoPC present in the samples. Note that in rat, as described for human, lysoPCs 16:0 and 18:0 are the most abundant species found in plasma[16,39]. Inset: Typical MS spectra from plasma lipid extract showing identified lysoPC species and internal standards (i.s.). m/z = mass/charge ratio.

Lipids were extracted from plasma samples using the Folch method[32] and total phospholipids (PL) were determined[33]. Samples were mixed with a fixed amount of insect derived. N-Acetyl-L-erythro-sphingosine ("internal ceramide", from Sigma) as a control, dried under nitrogen and re-suspended in chloroform before spotting on Merck 5631 TLC plates. The plates were developed in 3 directions with drying between each migration[34]. Solvents for the first and second migration were 1) 60:30:8:2, chloroform: methanol: formic acid: H20, and 2) 50:40:7:3, chloroform: methanol: 28% ammonium hydroxide, respectively. For the third migration the plates were developed in diethyl ether. After development, the plates were immersed in 10% CuSO_3 _and 8% H_3_PO_4 _solution for 20 seconds, and charred at 180°C for 20 min [35]. The total intensity of charred spots (area × mean optical density) was determined with Image J (public domain software) and values were compared to standard curves (Fig. 1B) obtained with authentic standards from Avanti Polar Lipids (Alabaster, AL).

#### Mass Spectroscopy

Samples from the Folch-extracted lipid were dried and re-dissolved in chloroform-methanol-300 mM ammonium acetate in water (60:133:7, v/v/v). Lysophosphatidylcholines were analyzed by electrospray ionization triple quadrupole mass spectrometry at the Kansas Lipidomics Research Center, as previously described[36].

### Enzyme assays

Enzyme assays were conducted as in previous studies [23] using a Victor 3 fluorescent reader (Perkin Elmer, Waltham, MA). The substrate was 1-palmitoyl-2-pyrenedecanoyl phosphatidylcholine ("10-pyrene"), Caymen Chemical, Ann Arbor MI) a substrate for all calcium dependent PLA2s with the exception of cPLA2 and PAF-AH (LP-PLA2). Substrate solutions were prepared in reaction buffer consisting of 50 mM tris (pH = 7.4), 0.1 M NaCl, 2 mM CaCl_2_, 0.25% fatty acid-free albumin. The substrate forms phospholipids vesicles in this solution [37], and upon hydrolysis, releases fluorescent 10-pyrenyldecanoic acid (PDA). Plasma samples were 10% final and mixed with lysoPCs by gentle shaking for 100 sec, or they were derived from lysoPC-injected rats. All enzyme reactions were initiated with the addition of the substrate solution to the sPLA2 containing samples. Kinetic parameters including the properties of lysoPC inhibitors were determined by recording the initial maximum velocities (Vo) of enzyme reactions, obtained within 2 minutes of initiation. A detailed presentation of the calculations used in our kinetic analysis has been presented [23]. Relative fluorescent units (RFU) were converted to product concentration using a standard curve made with pyrenyldecanoic acid (Invitrogen, Eugene, OR). Individual reactions were carried out in duplicate or triplicate and kinetic curves were produced using at least 6 substrate concentrations, with lysoPC or its vehicle (PBS with 8% ethanol). Representative Lineweaver-Burk and Michaelis-Menten plots as well as nonlinear regression analyses are presented in Results. Experiments were repeated at least 4 times with similar results. Statistical analyses were by the Mann-Whitney test or Welch test for the *in vivo *data since control data for the latter was Gaussian with a variance that was significantly different from the experimental samples. These calculations, and Km, Vmax and R^2 ^were made with statistical (INSTAT) and regression software (Prism), both from Graphpad (San Diego, CA).

### LysoPC treatment of rats

Rats were injected subcutaneously under the loose skin at the shoulders with synthetic stearoyl (n = 4) or palmitoyl (n = 4) lysoPCs (25 mg/kg) two hrs before sacrifice and compared to vehicle-injected rats (n = 8). The relative concentrations of active sPLA2 enzymes were measured in plasma samples of these animals by determining the total hydrolysis of substrate in 1 hr.

## Results

### Plasma lysophosphatidylcholines

The two-dimensional thin layer chromatography procedure we adapted was developed to reveal several of the most abundant phospholipid and sphingolipid species on a single TLC plate [34] (Fig. 1A). Phosphatidylcholine, sphingomyelin and lysophosphatidylcholine, were the major phospholipids appearing on the plates with plasma samples. Ceramides, cholesterol, free fatty acids, and phosphatidylinositol also appeared in significant quantities while lower levels of phosphatidylethanolamine and galactoceramide were found with this procedure. Therefore, these plates showed that lysoPCs were a significant proportion of the lipid component of blood plasma in rodents and humans, as reported by others [16,38-43]. However, published reports or calculations made from published reports provide a fairly wide range of estimates of the total lysoPC concentration in plasma (145 to 270 μM). Our estimates of total lysoPC, including from standard curves generated on the TLC plates (Fig. 1B), were 207.5 ± 44 μM (TLC, n = 3) and 196 ± 64 μM (mass spectroscopy, n = 3). Mass spectroscopy analysis also showed that palmitoyl (16:0) and stearoyl (18:0), calculated as mol percent of total lysoPC present, were the most abundant species in plasma, followed by linoleoyl (18:2), and oleoyl (18:1) lysoPCs, in general agreement with the previous studies cited above, and similar for rats and humans (Figs. 1C, D).

### Inhibition of sPLA2 activity

#### Plasma

**Figure 2:**
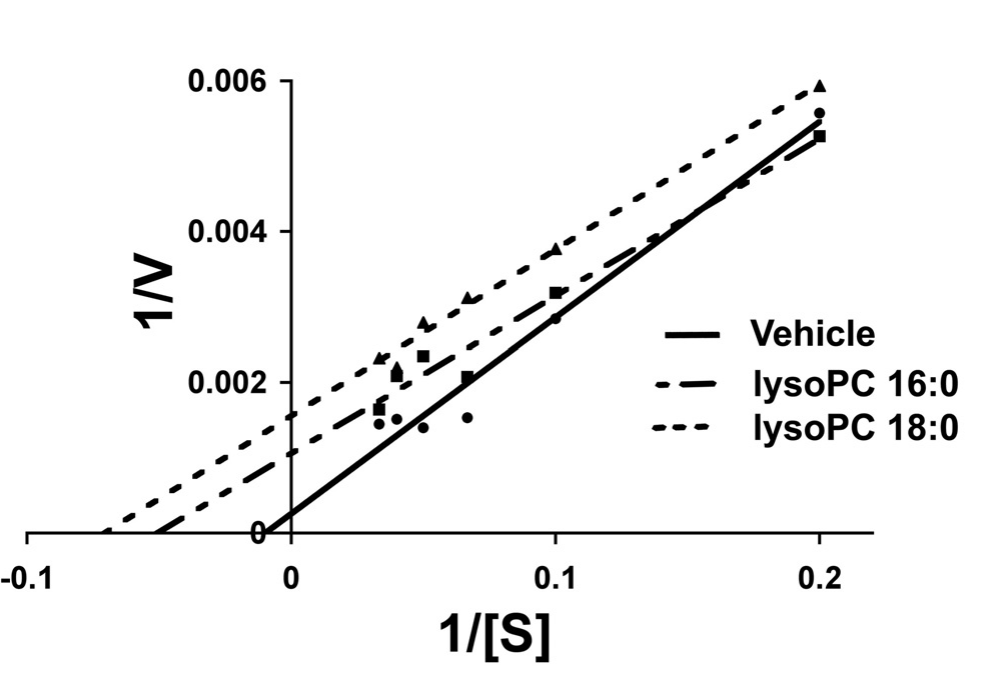
**Lineweaver-Burk double reciprocal plots illustrating the kinetics of plasma sPLA2 activity in the presence of lysoPCs 16:0 and 18:0**. Plots are from *ex vivo *experiments with plasma. More inhibition (larger differences in 1/V) was found with added lysoPCs (20 μM) as the substrate concentration was increased (smaller 1/[S]). LysoPC treatment also reduced Km (increase in the absolute value of the x intercept). These kinetic parameters were also calculated by nonlinear regression of Michaelis-Menten functions and confirm the uncompetitive character of the inhibition (Table 1).

The possibility that there are endogenous sPLA2 inhibitory factors in plasma was suggested in a previous study because spontaneous inhibition of the enzyme reaction was observed in plasma samples as the concentration of substrate was increased [23]. At these points of higher activity, the fit to a classic hyperbolic function had deteriorated. In the present study therefore, we attempted to minimize the contribution of endogenous factors by using plasma samples and substrate concentrations where the enzyme reaction still followed Michaelis-Menten kinetics (Table 1). Under these conditions, the influence of endogenous inhibitory factors was expected to be small (see also below). We tested the effects of adding lysoPCs 18:0 and 16:0 to these plasma samples prior to initiating the enzyme reaction. We used synthetic LysoPCs to be certain of the fatty acid composition and therefore the purity of the species tested. Both 18:0 and16:0 lysoPCs showed consistent inhibition of sPLA2 activity (Fig. 2). These data showed that the extent of the inhibition depended on substrate concentration and therefore was likely to be uncompetitive. The kinetic data derived from the regression analysis of the Michaelis-Menten curves confirmed this, showing reductions in both Vmax and Km, the defining feature of uncompetitive inhibition (Table 1).

**Table 1:** Kinetic data for lysoPC inhibition of sPLA2 activity in plasma

**Plasma Treatment**	Vmax (nM/min/ml)	Km (μM)	R^2^	Ki ± s.e.m. (μM)
**Vehicle**	1296	33.24	0.9938	
**Palmitoyl**	684.7	16.12	0.9595	25.9 ± 4.0
**Vehicle**	1395	27.24	0.9413	
**Stearoyl**	669.2	11.26	0.928	34.2 ± 17.4

Data from enzyme reactions of plasma samples containing lysoPC (20 μM) or vehicle. The reactions for each lysoPC–containing sample were run side by side with vehicle-containing samples and they were drawn from the same pool of plasma. In each case the lysoPC reduced both Km and Vmax showing the inhibition was uncompetitive. Average Ki values with s.e.m. were calculated as in previous studies from all reactions with substrates above 15 μM [23]. R^2 ^= fit to Michaelis-Menten function.

#### Group IA sPLA2

**Figure 3:**
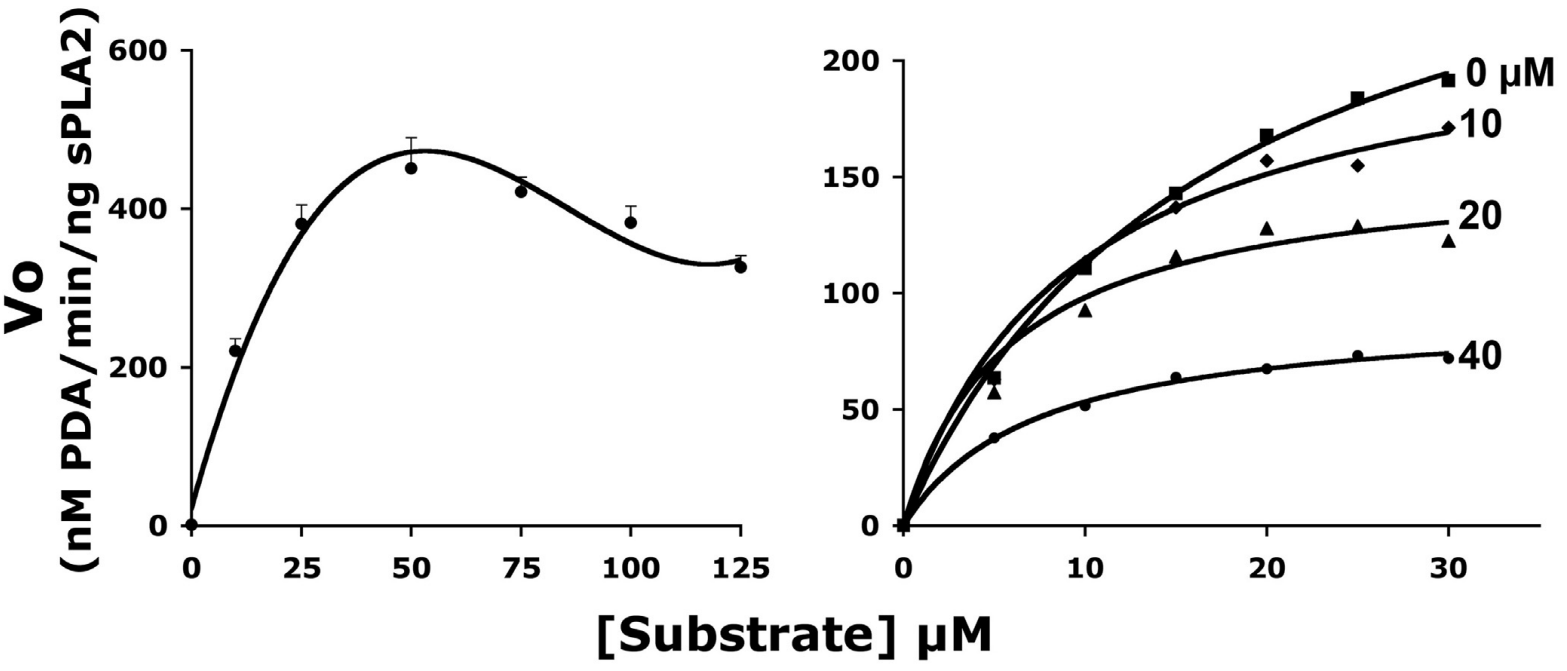
**LysoPC inhibition of group IA sPLA2 activity**. (A.) Relationship between substrate concentration and velocity in reactions of group IA sPLA2 in the presence of high levels of substrate but without inhibitor. The data suggested there was endogenous inhibition at the highest substrate concentrations as the plot deviated from the typical hyperbolic curves shown in B. (B.) Plots of Vo versus substrate concentration for group IA sPLA2 activity in the presence of different concentrations of lysoPC 18:0. In this lower range of substrate concentrations, typical hyperbolic curves were obtained. Note that progressive inhibition of activity was found up to 40 μM lysoPC. All reactions were with 15 nM sPLA2. The kinetic parameters for these reactions calculated by nonlinear regression are shown in Table 2: PDA-pyrenyldecanoic acid.

Plasma is a complex fluid and therefore presents endless possibilities for non-specific or unexpected interactions with the enzyme reactants. Therefore, we repeated the above experiments *in vitro *using potent venomous group IA sPLA2. The purpose of these experiments was also to re-examine product inhibition of sPLA2 activity, especially in relation to albumin concentration, already reported for group IA sPLA2[30,31]. The possibility of endogenous inhibition in these reactions was examined first with elevated concentrations of substrate. It is important to note that one product of reactions with the 10-pyrene substrate is palmitoyl lysoPC which is a potential inhibitor. In fact, Vo declined at the highest substrate concentrations resulting in kinetic curves that showed the best fit to a third order polynomial (R^2 ^= 0.9821) rather than a hyperbolic function (Fig. 3A). These complex kinetics and the fall off of activity could have been due to product inhibition. In order to test this proposition further, we again took the approach of reacting the enzyme with a lower range of substrate concentrations (where the activity still followed Michaelis-Menten kinetics) but with added lysoPCs (Fig. 3B). Uncompetitive inhibition was observed with both the 16:0 and 18:0 species. Representative Michaelis-Menten plots, obtained for four concentrations of the lipid, are shown for lysoPC 18:0 (Fig. 3B). Regression analysis of the data again showed a reduction in Vmax and Km (Table 2). However, lysoPC 10:0, which was not detected in plasma and therefore was a lipid class specific control, failed to show inhibition tested under conditions that gave maximal inhibition with the other lysoPC species (i.e. 40 μM lysoPC, 30 μM substrate, Table 2).

**Table 2:** Kinetic data for lysoPC inhibition of group IA sPLA2

**[LysoPC] μM**	Vmax (μM/min/ml)	Km (μM)	R^2^	Ki ± s.e.m. (μM)
**Stearoyl**				
**Vehicle**	305.8	17.12	0.993	n.c.
**10**	221.9	9.37	0.959	n.c.
**20**	156.2	5.92	0.808	n.c.
**40**	92.15	7.31	0.949	14.46+1.15
**Decanoyl**				
**Vehicle **	355.7	23.76	.984	-----
**40**	342.4	25.00	.979	-----

The kinetic parameters for enzyme reactions containing 0–40 μM lysoPC 18:0 (stearoyl), and 15 nM group IA sPLA2 (see Fig. 3). Vehicle = no lysoPC. As the concentration of lysoPC increases Vmax is reduced. This result, along with the consistent reduction in Km, demonstrated that the inhibition was uncompetitive. The Ki value was calculated for 40 μM lysoPC only and from reactions with substrates above 15 μM (n.c. = not calculated). R^2 ^= fit to Michaelis-Menten function. No inhibition was found after addition of 40 μM lysoPC 10:0 (decanoyl) to the reactions containing 30 μM substrate when compared to vehicle controls reacted at the same time.

#### Albumin influences

Albumin binding by the lysoPCs may influence some of their biological activities (see Discussion), but of special interest in the present study was the possibility that albumin also neutralizes group IA sPLA2 product inhibition *in vitro *[30,31]. In the present *ex vivo *experiments with plasma, significant inhibition sPLA2 activity was observed even though albumin is major constituent of plasma (approximately 3% or ~400 μM) [44]. Since one albumin molecule can bind two lysoPC molecules [30], and given the estimated concentrations of lysoPCs in plasma (see above), it appears that all lysoPC molecules with a propensity for albumin binding could be bound. It is suggested therefore that albumin does not influence lysoPC inhibitory activity at least under physiological conditions. We observed attenuated inhibition of group IA sPLA2 with albumin, but only after preincubation of 40 μM lysoPC 18:0 with a large excess of this protein (5% which is > 600 μM). In these cases, the percent inhibition (i.e., the percent reduction in Vo) was 29.3 ± 1.6% (n = 4) compared to 63 ± 6% (n = 8) with standard buffer (p < 0.01). This difference was significant (p = 0.029).

#### LysoPC treatment of rats

**Figure 4:**
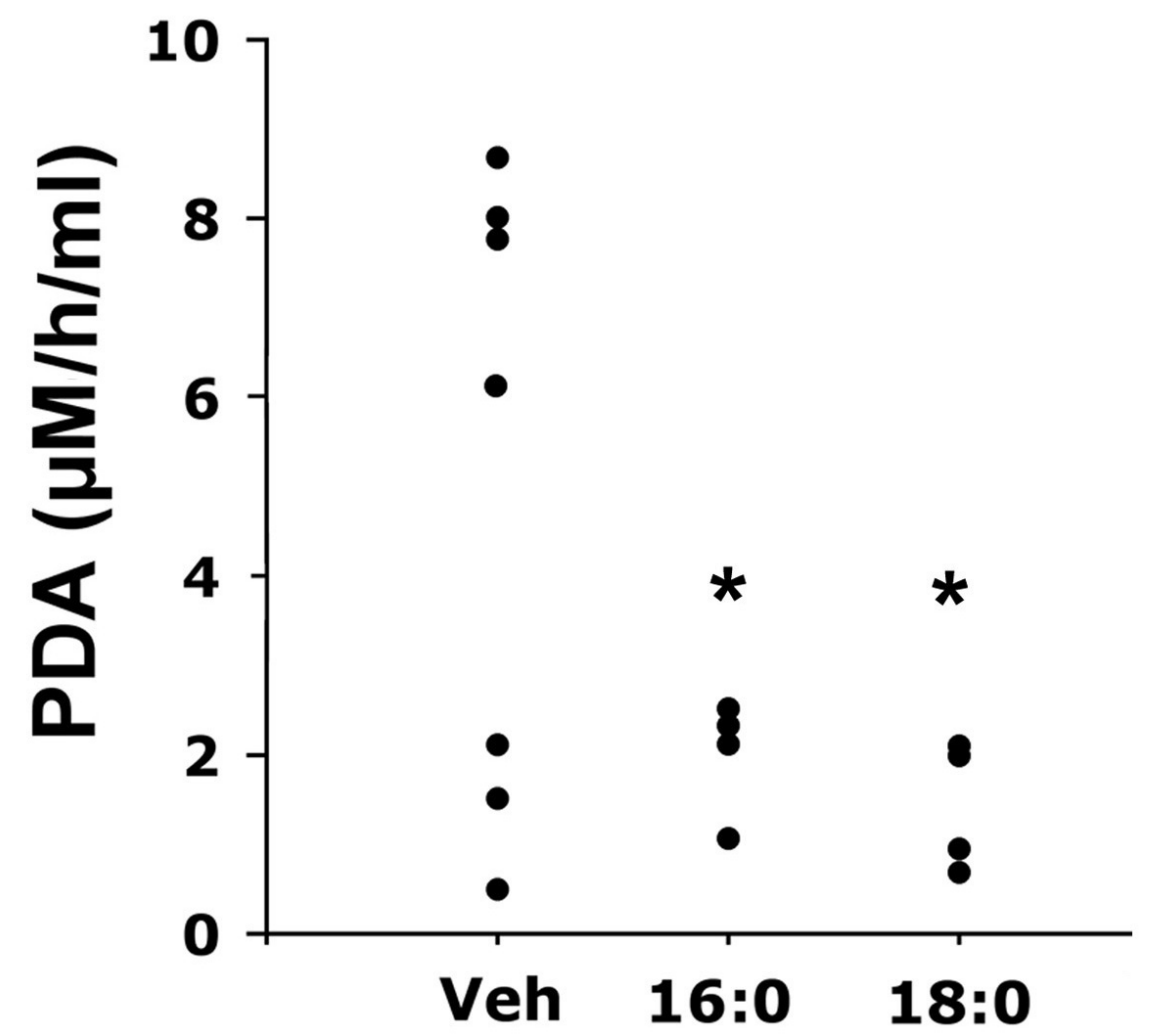
**LysoPC treatment of normal rats**. Twelve rats were treated with subcutaneous injections of 0.25 mg/kg lysoPC in PBS containing 8% ethanol or PBS-ETOH vehicle only. The rats experienced mild restraint during the injections. Two hours later, the rats were sacrificed and plasma sPLA2 activity was measured by determining the amount of pyrenyldecanoic acid (PDA) generated from the 10-pyrene substrate in one hour. A typical spread of enzyme activities was seen in the vehicle-treated rats while lysoPC treatment produced a range of activities that were significantly narrower and lower. * = p < 0.05, Welch test.

Palmitoyl and stearoyl lysoPCs also regulated plasma sPLA2 activity 2 hrs after subcutaneous injections in rats (25 mg/kg). The approach to these studies was similar to that taken for the *in vitro *and *ex vivo *experiments since it was necessary to limit endogenous mechanisms of inhibition to interpret the results. Restraint and pre-measurement of activity, and/or experimentally elevating activity in individual rats, influenced the endogenous inhibitory mechanisms that were being studied [23]. In such cases, we were not successful in distinguishing between endogenous and exogenous inhibition, a pitfall that was not unexpected for experiments with naturally occurring uncompetitive inhibitors. Therefore, measurements were made on lightly restrained vehicle-injected rats and showed a typical range of enzyme activities that were compared to those obtained from lysoPC- treated rats. Following lysoPC treatment, the range of sPLA2 activities was significantly lower and narrower than in control rats (Fig. 4). This restriction of enzyme activity was consistent with the uncompetitive inhibition demonstrated above.

## Discussion

The principal finding of this study is that the two most abundant lysoPC species in plasma are capable of inhibition of plasma sPLA2 activity. We documented this inhibition *ex vivo *and *in vivo *with plasma samples from rats after treatment with palmitoyl or stearoyl lysoPC species. The fate of these exogenous lysoPCs after they were added to the plasma or injected into the animals is uncertain. It is likely that they ultimately reside in one or more of the functionally available pools of lysoPC, either free, micellar, bound to LDL or serum proteins such as albumin or immunoglobulin (anti-phospholipid-immune complexes). The relative abundance of these forms and of the total lysoPC concentration in plasma is expected to be highly regulated, including by the balanced activity of sPLA2s and lysophosphatidylcholine acyltransferases [45], enzymes that cleave and attach the A2 fatty acid. Despite these uncertainties as to the fate of the exogenous lysoPCs, it appears that a 10–20% increase in their total molar concentration in plasma is sufficient to inhibit sPLA2 activity *ex vivo*. In addition, the fact that we used a general sPLA2 substrate further suggests these lysoPCs have the potential to inhibit several of the sPLA2 isoforms found in plasma [46,47].

**Figure 5:**
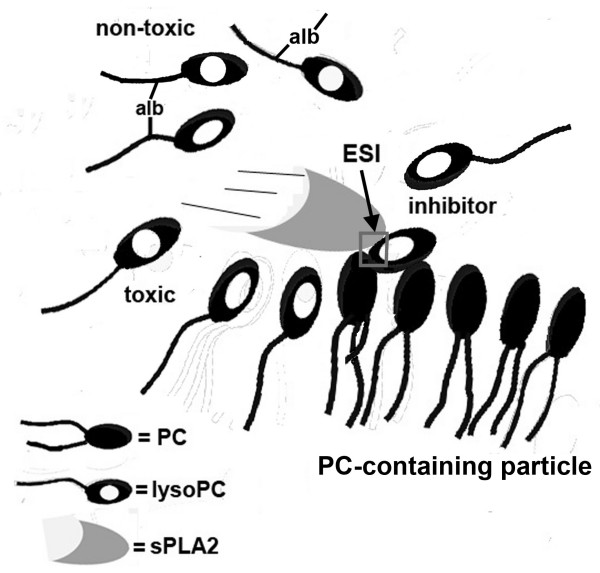
**Schematic illustrating albumin bound and free LysoPCs relative to circulating phosphatidylcholine (PC)-containing particles and sPLA2 enzymes**. According to current theory, the sPLA2 enzyme "scoots" along the PC vesicle cleaving the A2 fatty acid to produce lysoPC (see text). The desorbed or free lysoPC may be toxic and but this toxicity is neutralized by albumin binding. It is suggested that when excessive lysoPCs are produced during periods of high enzyme activity, they become sPLA2 enzyme inhibitors acting at the point of advancing PC hydrolysis. This configuration would allow for the formation of the Enzyme-Inhibitor-Substrate (ESI) complex, a feature of the uncompetitive inhibition demonstrated in this study. Exogenous lysoPCs may augment this process. It is unclear whether the lysoPC acting as an inhibitor is bound to albumin, but in the present experiments, physiological concentrations of albumin had no discernable effect on the inhibition.

Uncompetitive inhibitors are ideal for *in vivo *applications because they are most effective during periods of elevated enzyme activity, but do not necessarily respond to normal levels of activity [23,48,49]. These inhibitors bind the enzyme-substrate complex (ES), and for sPLA2 enzymes, levels of this complex are highest during periods of inflammatory stress [23]. Since there are presumably high levels of lysoPC product at this time, all the elements required to optimize uncompetitive inhibition are well represented as the sPLA2-driven inflammatory response proceeds. This uncompetitive property of the inhibition also suggests the biomolecular configuration of the inhibitory complex, i.e., where this inhibition is likely to occur. Assuming the enzyme "scoots" along the surface of circulating phospholipid (PL) particles [50], it is suggested that the lysoPC inhibitor attacks the substrate at points of advancing enzyme activity (Fig. 5). It would be at these points that enzyme, substrate, and lysoPC inhibitor are adjacent and could form the enzyme-substrate-inhibitor (ESI) complex. The lysoPC involved in the inhibition could come from the hydrolysis of PC containing particles ("desorbed" lysoPC) [51], free, or albumin-bound lysoPC in the circulation (see below), or from exogenous sources as in our experiments.

The pro-inflammatory effects of lysoPCs have been well documented, both as exogenous agents of toxicity and as products of sPLA2 hydrolysis. For example, the contribution of lysoPCs to atherosclerotic lesions is a prime example of ability of sPLA2 enzyme activity to direct a local injurious inflammatory response. LysoPC has been considered a principal agent of this activity [52,53]. In the nervous system, exogenous lysoPCs are used in demyelination studies and as a trigger for responses of the innate immune system in order to model neuroinflammatory disorders [54]. Glutamate toxicity after cerebral ischemia is exaggerated by local infusion of lysoPC [55], an effect attributed to the "detergent action" of the lipid [56]. In the plasma however, where most lysoPCs are bound to albumin [39,43,57], it is assumed that endogenous lysoPCs are much less toxic, perhaps because of this binding [58]. Our data indicate that at physiological concentrations, albumin does not effect lysoPC inhibition of plasma sPLA2 activity *ex vivo *or *in vivo*. Considering these results and the model presented above, it is can be suggested that: 1) albumin binding does not effect the binding to PC containing particles and subsequently the ability of the lysoPC to inhibit enzyme activity, or that 2), in some situations the affinity of the lysoPC for these particles is stronger than it is for albumin. The latter condition has been suggested to occur for oxidized low density lipoprotein (ox-LDL) particles both normally and during certain pathologies [57,59]. One interesting possibility is that the affinity of the PC-containing particles for lysoPCs is regulated by sPLA2 activity; for example, as activity is increased, the concentration of desorbed lysoPCs also increases, making the particles more receptive to incorporating the lysoPC product, which in turn limits enzymatic activity. The present results suggest that exogenous lysoPCs also increase this affinity, thereby accomplishing sPLA2 inhibition and cytoprotection. In this circumstance however, the potential toxicity of excess lysoPCs cannot be ignored, so there is likely an upper limit of lysoPC dosages that will accomplish the inhibition without cytotoxicity. Dose limitations of synthetic lysoPCs have been demonstrated for treatment of rodent endotoxaemia [17]. Along these same lines, the bactericidal properties of sPLA2s suggest a similar constraint exists regarding the extent of enzyme inhibition tolerated in models of fulminate bacterial infections [60].

The present results may complement recent studies that explore other mechanisms for the anti-inflammatory activities of lysoPCs. For example, the experiments of Chen, et al [20] showed the specific influence of LysoPC 18:0 on pro-inflammatory HMGB1 release from monocytes and macrophages. Although this effect appears partially mediated by the G2A receptor, we suggest that the well-known relationship between sPLA2 activity and macrophage activity is also relevant, especially because of the late stage at which the lysoPC was administered [2,45,61,62]. It is expected that during this late period, the cascading inflammatory response would make conditions ideal for uncompetitive product inhibition of sPLA2 and resulting attenuation of monocyte/macrophage functions. Likewise, there is substantial cross talk between sPLA2 enzymes and the activity of pro-inflammatory cytokines that have been implicated in sepsis models [17]. The suggestion that lysoPC 18:0 also enhances microbial elimination during sepsis induced by cecal ligation and puncture [18] might reflect direct effects of the lysoPCs on bacterial sPLA2 enzymes, since bacterial sPLA2s with remarkable similarities to the mammalian enzymes have now been characterized [63]. Finally, the diminished LysoPC/PC (product/substrate) ratio found in sepsis patients that are more likely to recover [16] is entirely consistent with inhibition of sPLA2 activity described here. The inhibition might slow the inflammatory cascade and increase the probability of recovery.

## Conclusion

The diverse, sometimes contradictory properties of LysoPCs suggest that the different functional forms of this class of lipids are highly regulated in the plasma. Under conditions of severe inflammatory stress and subsequent elevation of plasma sPLA2 activity, systemic lysoPCs will help to shift the balance towards sPLA2 inhibition and cytoprotection. LysoPC therapies are therefore possible if these lipids are introduced systemically at the appropriate levels and at the appropriate point in the inflammatory cascade.

## Competing interests

The authors declare that they have no competing interests.

## Authors' contributions

All three authors were involved in different aspects of the enzyme analyses. LY and AL were responsible for the lipid analyses. TJC wrote the original draft of the paper after which it was reviewed, modified, and corrected by the other authors.
